# Age-Related Deficits in Spatial Memory and Hippocampal Spines in Virgin, Female Fischer 344 Rats

**DOI:** 10.1155/2011/316386

**Published:** 2011-08-18

**Authors:** Victoria N. Luine, Maureen E. Wallace, Maya Frankfurt

**Affiliations:** ^1^Department of Psychology, Hunter College, CUNY, New York, NY 10065, USA; ^2^Biopsychology and Behavioral Neuroscience Program, The Graduate Center, CUNY, New York, NY 10016, USA; ^3^Department of Science Education, Hofstra North Shore-LIJ School of Medicine, Hempstead, NY 11549, USA

## Abstract

Effects of aging on memory and brain morphology were examined in aged, 21-month-old, and young, 4-month-old, Fischer 344 female rats. Spatial memory was assessed using the object placement task, and dendritic spine density was determined on pyramidal neurons in the hippocampus following Golgi impregnation. Consistent with previous studies, aged females showed poorer object placement performance than young subjects. Young subjects significantly discriminated the location of objects with a 1.5-hour intertrial delay while aged subjects did not. Spine density of basal dendrites on CA1 pyramidal cells was 16% lower in the aged subjects as compared to the young subjects. No differences in spine density were found between young and aged subjects in basal dendrites of CA1 or in either dendritic field of CA3 pyramidal neurons. Thus, decreased hippocampal CA1 dendritic spine density in aged rats may contribute to poorer spatial memory as compared to young rats. The possibility that the neuroplastic changes observed in this study may pertain only to female subjects having had a specific set of life experiences is discussed. Different factors, such as reproductive status, diet, and handling may contribute to neuroplasticity of the brain during aging; however, this view requires further examination.

## 1. Introduction

It is well established that aging in animals and humans is accompanied by a decline in the ability to acquire and remember information. In rodents, spatial memory tasks that require the hippocampus show reliable performance decrements across the lifespan [1–3]. Although most studies have examined males, the few studies done in females also show age-related performance declines. Aged female rats, approximately 21–24 months old, show impairments, as compared to 2–4-month-old young rats, on a number of spatial memory tasks including Y and T mazes [4], radial arm maze [5, 6], Morris water maze [1, 7], water radial arm maze [8], and Barnes maze [9]. However, the specific mechanisms responsible for age-related declines in spatial memory remain largely unclear [10]. 

The hippocampus has been shown to undergo age-related physiological and morphological changes which may provide the basis for the observed deficits in cognitive function, especially spatial memory [11]. Since overall loss of neurons does not appear responsible for the behavioral changes [3, 12] other neuroplastic changes, which have been shown to correlate with altered neural functions associated with learning and memory, have been examined. Thus, spine density and synapse number have been compared in young and aged rodents, mainly males, as possible mechanisms contributing to impaired spatial learning and memory during aging. However, there are few studies and the results are inconclusive. Using Golgi-impregnation, Markham et al. [13] and, Alcantara-Gonzales et al. [14] found that there were no changes in spine density in the CA1 hippocampal region in 19–22 or 24–26-month-old Long Evans males as compared to 3–5 or 12-month-old rats, respectively. In contrast, Calhoun et al. [15] found a modest increase in spine number when staining for the spine-associated protein, spinophilin, in the CA1 region of the hippocampus in 24–26-month-old Long Evans male rats as compared to 8-month-old rats. In a Golgi impregnation experiment in mice of both sexes, Von Bohlen et al. [16], reported decreased CA1 basal spine density in 21-22-month-old as compared to 6-7-month-old mice. Markham et al. [13], examining only females, showed that 19–22-month-old retired breeder, Long Evans rats did not have altered CA1 spine density as compared to 3–5 month old females. This general lack of agreement in findings on neuroplastic changes in the hippocampus with aging has been ascribed to possible sex and strain differences, but few studies have examined females [12, 13], and not all strains have been systematically compared. Moreover, only a few studies have examined both the morphology of the hippocampus and spatial memory in aged and young subjects, and these have only been completed in male subjects [15, 17] or with no regard to sex [16]. 

In the present study, we investigated both morphology and memory in aged (21 months old) and young (4 months old) virgin, female, Fischer 344 rats. Subjects were tested for spatial memory using the object placement task [18], a relatively new, working memory task the performance of which has not yet been assessed in aged versus young rats. One week after spatial memory testing, spine density was assessed in hippocampal pyramidal neurons using Golgi impregnation to determine whether possible age-related declines in spatial memory are associated with changes in spine density in CA1 and CA3 hippocampal pyramidal neurons.

## 2. Experimental Procedures

### 2.1. Subjects

Ten 19-month-old and eight 2-month-old virgin female Fischer 344 rats were obtained from the National Institute of Aging Colony at Harlan Sprague-Dawley, Inc. The experiment was approved by the Hunter College IACUC and carried out in accordance with the National Institute of Health Guide for the Care and Use of Laboratory Animals. Rats were double housed and maintained on a 12-hour light/dark cycle (lights off 7:00 pm) with food and water *ad libitum*. The chow used was Harlan 2016 Teklad Global 16% Protein Rodent Diet chow which is low in phytoestrogens. Standard rat chows, like Purina LabDiet chow, contain approximately 810 *μ*g/g of phytoestrogens, derived mainly from soymeal [19] while the 2016 formula contains no soy or alfalfa, and therefore contains minimal phytoestrogens (see http://www.teklab.com/). This special diet was used because we have previously reported that ovariectomized (OVX) rats chronically fed chow containing high phytoestrogen levels, as compared to minimal levels, show significantly better object placement performance and have increased dendritic spine density in CA1 and prefrontal cortex pyramidal neurons [20]. Since aged female rats also have low estradiol levels, it is possible that a diet high in phytoestrogens might affect them in a manner similar to that of OVX rats. Subjects were allowed two weeks to acclimate to new housing and were then handled daily for an additional two weeks prior to beginning of experimentation in order to minimize possible effects of stress on performance and brain function.

### 2.2. Memory Assessment

Spatial memory was assessed using the object placement task as previously applied in young rats and recently described in detail [21]. All trials were conducted in an enclosed arena measuring 65 × 65 × 30 cm, with an open top. Subjects received extensive habituation trials in order to minimize effects of stress and/or anxiety to the field or to the objects on memory. Trials were initiated with a 5 min session on the arena without objects, and then habituation to the objects began. Trials consisted of a sample trial (T1) and recognition trial (T2) which were separated by an intertrial delay. During habituation trials, the intertrial delay was increased each day. In T1, two identical objects were placed at one end of the arena and time spent exploring the two objects was recorded for 3 min. For T2, one new, novel object was substituted for one of the objects. Trials with 10 sec, 30 min, 1 h, and 2 h intertrial delays were given on consecutive days. Time spent exploring the new versus the old object was recorded for each subject during T2. As reported previously for Fisher 344 rats, aged subjects significantly discriminated between old and new objects only for the 30 min intertrial delay whereas young rats significantly discriminated at 30 min, 1 h, and 2 h intertrial delays [22]. Then subjects were habituated for place trials. T1 was identical to the object recognition trials, but in T2 one object was moved to a new location, and time spent exploring objects at the old versus the new location was recorded for each subject. Subjects received habituation trials with a 1, 15, and 40 min intertrial delay. Thus, a total of two weeks of habituation trials to the task was given. Then subjects received testing for object placement. Three trials with 1, 1.5, and 2 h intertrial delays were given. The percentage time spent with the object in the new location during T2 was used as an index of object placement performance (sec at new/sec at new + sec at old). The new location was counterbalanced within groups, and the field and objects were cleaned with Bacdown Detergent Disinfect spray both between T1 and T2 for individual animals and between separate trials for each subject. Objects included ceramic, glass, and metal figurines (average weight 200 gm and height 5–10 cm) and new sets of objects were provided for each trial. One aged subject did not explore in a sample trial and was not entered into the behavioral or morphological dataset.

### 2.3. Golgi Impregnation and Analysis

**Figure 1:**
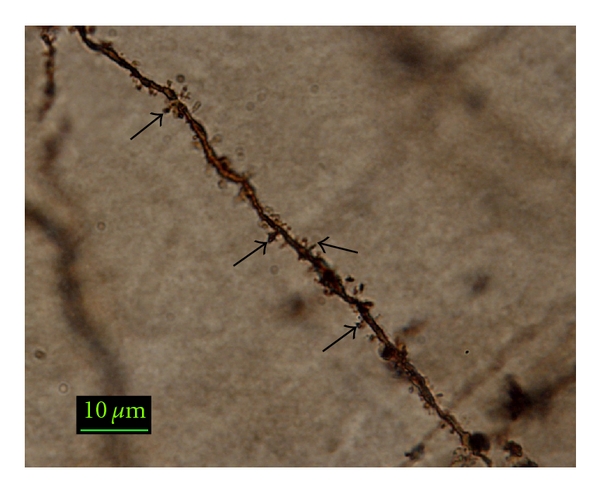
Photomicrograph (100x) of a secondary basal dendrite from a CA1 pyramidal cell taken under oil immersion from a young female. Arrows denote spines.

One week after completion of behavioral testing, at 4 and 21 months of age, rats were sacrificed and brains processed for analysis using the FD Rapid GolgiStain kit (FD NeuroTechnologies, Inc) as previously described [20, 23]. We waited a week before sacrifice because it has been reported that performing an associative memory task increases dendritic spine density in basal dendrites of the CA1 region [24]. Blocks of brain tissue containing the hippocampus were made by cross-sectional cuts of the brain at the level of the optic chiasm and mammillary bodies. Blocks were rinsed in 0.1 M phosphate buffer and then immersed in the commercial kit combination of potassium dichromate and mercuric chloride solution for 14 days. Brains were then transferred to a sucrose-based solution and stored at 4°C for 2–5 days. The tissue blocks were sliced into 100-*μ*m sections in a cryostat and mounted on gelatin-coated slides. Following sectioning, a drop of sucrose solution was placed on each of the sections on the slides and the excess absorbed off with filter papers. Then slides were dried at room temperature. When dry, sections were rinsed with distilled water and placed in solution containing silver nitrate for 10 min. Following impregnation, they were rinsed in distilled water, dehydrated in 50, 75, 95, and 100% ethanol and then cleared in Protocol (Fisher Scientific) and cover slipped in Permount. See Figure 1 for a representative, Golgi-impregnated neuron from a basal CA1 dendrite in a young subject. 

For spine density analysis, secondary basal dendrites and tertiary apical dendrites from CA1 and CA3 hippocampal pyramidal cells were counted using the Spot Advanced program, version 3.5 for Windows (Diagnostic Instruments, Inc) and a Nikon Eclipse E400 microscope. Dendrites used had to meet the following criteria: the cell body had to be in the area of interest, the length of the branch could not be broken, and the length of the dendritic branch had to be isolated well enough for unobstructed views. Finally, for consistency, the most lateral dendrite was always used. For a given length of dendrite, the spines were counted by two different investigators (blindly) and the mean of the two counts obtained were used to calculate the spine density. Spines were counted under oil (100x), going in and out of focus, to assure that all the visible spines were counted. The length of the dendrite counted was then measured using the Spot Image Analysis system. Five to six cells/brain were averaged and spine density was expressed as spines/10 *μ*m dendrite.

### 2.4. Serum Estradiol Measurement

At sacrifice, trunk blood samples were collected for measurement of serum estradiol using radioimmunoassay (Coat-A-Count, kit KE2D 1, Diagnostic Products Corporation, Los Angeles, CA) as previously described [25].

## 3. Results

### 3.1. Spatial Memory

**Figure 2:**
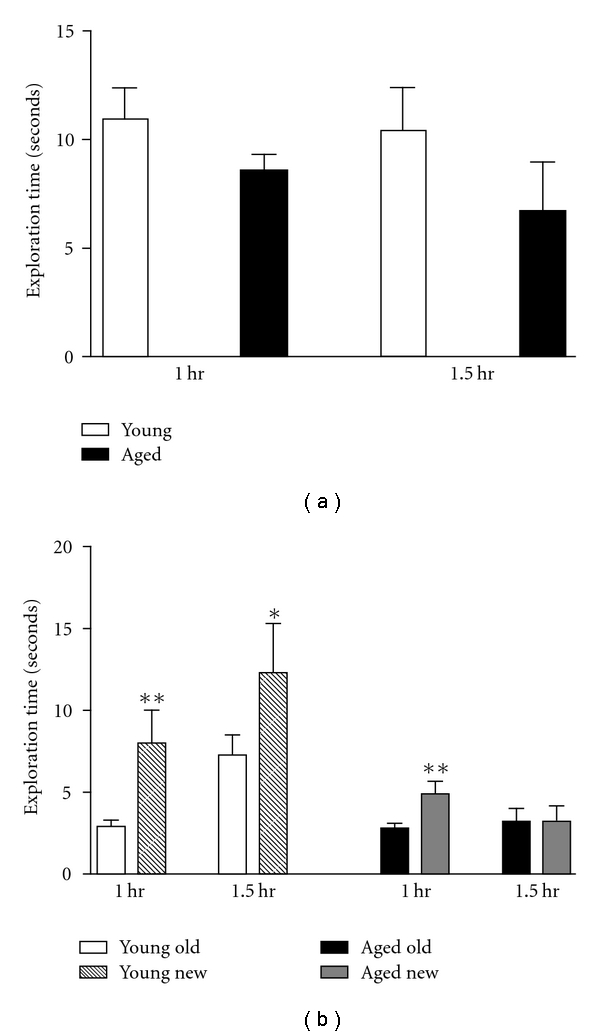
(a) Object placement-exploration time during the sample trial. Mean exploration time ± SEM for young (*n* = 8) and aged rats (*n* = 9) prior to 1- and 1.5-hour intertrial delay trials. No significant differences by two-way ANOVA (group × trial). (b) Object placement-exploration time during the retention trial. Mean exploration time ± SEM at old and new locations is shown for 1- and 1.5-hour intertrial delay trials for young (*n* = 8) and aged (*n* = 9) rats. Data analyzed by three-way ANOVA, group × location × delay: group effect (*F*(1,76) = 19.36, *P* < 0.0001) indicated that the young group explored the objects more than the aged group; location effect (*F*(1,76) = 9.81, *P* < 0.002) and a group × location interaction (*F*(1,76) = 4.06, *P* < 0.04). Post hoc differences tested by paired *t*-tests where *P < 0.05, **P < 0.01.

Subjects were tested on the object placement task with intertrial delays of 1 and 1.5 h between the sample (T1) and the recognition trials (T2; Figures 2(a) and 2(b)). During the sample trials, there were no significant differences between groups in time spent exploring the objects (Figure 2(a), two-way ANOVA, group × trial). In the retention trials, exploration times were analyzed by three-way ANOVA (group × location × delay). A significant effect of group (*F*(1.76) = 19.36, *P* < 0.0001) indicated that the young group explored the objects more than the aged group. There were also effects of location (*F*(1,76) = 9.81, *P* < 0.002) and a group × location interaction (*F*(1,76) = 4.06, *P* < 0.04). Post hoc analyses (paired *t*-tests) tested whether young and aged groups significantly discriminated between objects at the old and new locations at each intertrial delay. At the 1 h intertrial delay, both groups discriminated between the old and new locations (young, *P* < 0.009; aged, *P* < 0.004, Figure 2(b)). At the 1.5-hour intertrial delay, only the young group significantly discriminated between the old and new locations (*P* < 0.04) suggesting an age-dependent impairment in this task. Neither group significantly discriminated with a 2-hour intertrial delay (data not shown), a result which is consistent with previous studies showing that young female rats cannot discriminate at long intertrial delays of the object placement task [26, 27]. 

### 3.2. Dendritic Spine Density

**Figure 3:**
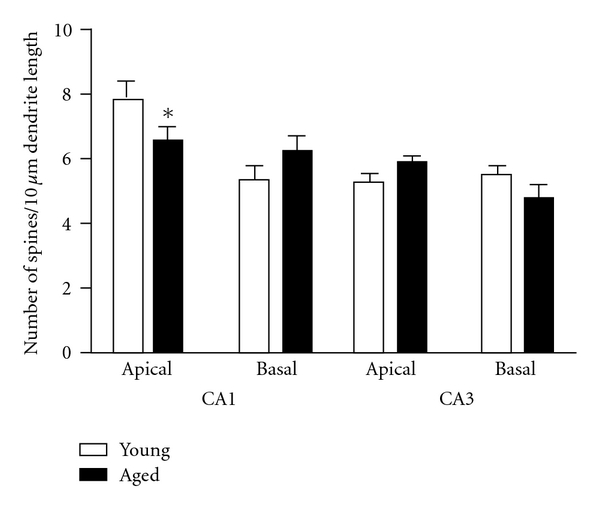
Spine density. Columns are the mean number of spines/10 *μ*m of dendrite length ± SEM. Data analyzed by two-way ANOVA, group (young, aged) × field (CA1 apical, CA1 basal, CA3 apical, CA3 basal). A significant effect of field, *F*(3,60) = 14.24, *P* < 0.000, and a significant group × field interaction, *F*(3,60) = 3.35,   *P* < 0.009, was found. Post hoc differences between young and aged fields were tested by *t*-test, *P < 0.049.

Figure 3 shows spine density of apical and basal dendrites in pyramidal neurons of CA1 and CA3 of the dorsal hippocampus. Two-way ANOVA (group × dendritic field) indicated a significant effect of field, *F*(3, 60) = 14.24, *P* < 0.0001, and a group × field interaction, *F*(3, 60) = 3.35, *P* < 0.009. Post hoc *t*-tests showed that aged rats had significantly lower spine density (16%) in tertiary apical branches of CA1 as compared to young rats (*P* < 0.05). In contrast, no significant differences in spine density were found between groups for secondary basal branches in CA1 or in any field of CA3.

### 3.3. Estradiol Levels

**Figure 4:**
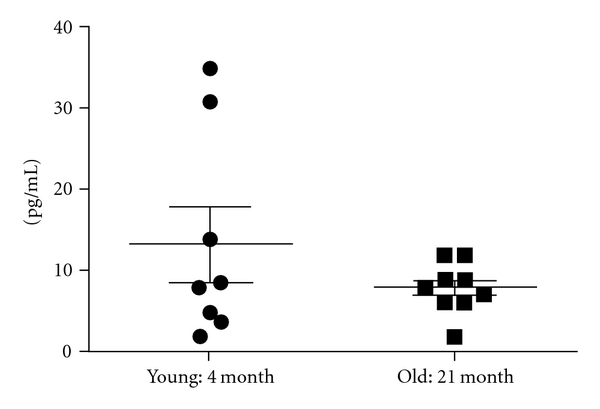
Serum estradiol. Entries show the average ± SEM and individual values for young (*n* = 8) and aged (*n* = 9) rats.

Serum estradiol (Figure 4) was 7.9 ± 1.0 pg/mL (range 2–12 pg/mL) in the aged subjects indicating a persistent diestrus state which is consistent with previous physiological observations of female rats at 21 months of age [13]. Young females had 13.5 ± 4.5 pg/mL of estradiol with a wider range in levels, 2–35 pg/mL, consistent with changes of the estrous cycle. Two young females had values in the 30 pg/mL range indicating the midproestrus stage of the estrous cycle, and the rest had values consistent with the diestrus or estrus phases of the cycle. 

## 4. Discussion

These results demonstrate that 21-month-old female rats show poorer spatial memory in the object placement task than do 4-month-old rats. In addition, the spine density of apical dendrites from pyramidal neurons in hippocampal subfield CA1 was reduced by 16% in the aged rats as compared with the young rats. In contrast, no differences in spine density were found in the basal dendrites of CA1 or in either subfield of CA3 pyramidal neurons. The behavioral data in the present study are consistent with a large body of aging research, but the morphological data provide novel information. 

The current study is the first to directly compare performance of aged and young female rats on the object placement task. The decrement in performance by aged, as compared with young rats, is consistent with results using other learning and memory tasks such as the radial arm maze [5, 6] and its water version [8] the Y and T maze [4], Barnes maze [9], and the most widely applied spatial memory task, the Morris water maze [1, 7]. Of note is that object placement requires little learning and is a working memory task while the other tasks are generally configured to measure both learning and memory [28]. Thus, these results show that memory alone is compromised in 21-month-old, aged female rats.

The significant decrease in spine density, 16%, in apical dendrites of CA1 pyramidal neurons, but not CA3 neurons, from aged female subjects as compared to young subjects is a novel finding. This change is, however, consistent with previously reported plastic changes in the hippocampus caused by gonadal hormones. CA1, but not CA3, spine density increases on proestrus during the estrous cycle [29] and following estradiol treatment to OVX rodents [30, 31]. We have also previously reported that OVX rats show a 17% decrease in CA1 apical spine density as compared to gonadally intact rats 7 weeks following OVX while there are no changes in the density of CA3 spines [23]. Aging in females is associated with a gradual cessation of ovarian steroid production, and the current aged subjects have reduced estradiol levels. Thus, the similar decrease in CA1 spine density in aged and OVX rats compared to young female rats suggests that ovarian hormones may be playing a role in the neuroplastic changes with aging. 

Hippocampal CA1 neurons are known to play a major role in spatial memory [3, 32], and thus, the decrease in CA1 apical spine density in aged females may contribute to age-related declines in spatial memory. However, few studies have examined memory function and spine density in the same subjects. We have reported that OVX subjects have decreased CA1 spine density as compared to gonadally intact rats and that these subjects also show impaired object placement memory [23]. Moreover, estradiol treatment to OVX mice enhances both object placement and CA1 spine density [31]. Von Bohlen et al. [16] also reported a decline in Morris water maze performance in a group of aged rats of both sexes that had decreased CA1 spine density. However, the number of axospinous synapses in CA1 was the same in young, aged-learning impaired, and aged-learning unimpaired male rats [17]. Thus, some, but not all, evidence supports the notion that CA1 spine density is important for maintenance of spatial learning and memory; however, further studies are necessary to establish whether a causal link exists.

While the current behavioral data are consistent with studies in aged rats, the morphological data in the present study differ from previously published results. Differences between the sexes may be important because the two studies in which Golgi impregnation was utilized demonstrated that spine density was not different in aged versus young male subjects, and these subjects were the same age or slightly older than the females in this study [13, 14]. Males do not undergo a large loss in gonadal hormones with aging which may account for the sex difference in the morphological change [33, 34]. However, strain differences cannot be discounted because subjects used in the Markham et al. study [13] were Long Evans rats while the rats used in the current were Fischer 344s. Our data is also different from the only other published study in females. Markham et al. [13], using Golgi impregnation, reported no changes in CA1 spine density in rats of a similar age as ours (19–22 mo.) but of a different strain, Long Evans. Nonetheless, it is notable that both groups are in agreement with reporting decreased spine density with aging in the cortex, cingulate, and prefrontal pyramidal neurons, respectively [23, 35].

We suggest that one important variable which may have contributed to our demonstration of decreased hippocampal spine density with aging is the reproductive history of our females. The Fischer 344 female rats used in the present study were virgins obtained from the National Institute of Aging Colony which may explain the differences between this and other aging studies in which retired Long-Evans breeding dams were used [13, 36]. Recent studies show that female rats that have been pregnant and reared pups (multiparous) generally show greater resilience to stress and have better memory abilities than female rats that have never experienced motherhood (virgin or nulliparous; see Macbeth and Luine [37]). For example, middle-aged rats (12 months old) that have had 4-5 pregnancies and births demonstrated better object placement performance and other memory tasks than age-matched, virgin rats [38]. Reproductive experience also apparently imparts long-lasting effects on memory processes because multiparous females show better spatial memory than age-matched virgins at 24 months of age [37, 39]. Moreover, pregnant or lactating females have greater spine density in CA1 than females at all stages of the estrus cycle [39, 40]. Whether pregnancy-related spine changes are as enduring as the memory changes is, however, unknown. Thus, use of rats with extensive motherhood and reproductive experience may be an important variable contributing to the lack of age-related neural changes in some previous aging studies utilizing female rats. 

Another factor which may have contributed to current neuroplastic effects during aging is that our subjects received a diet low in phytoestrogens. Typical rat chow contains these weak estrogens, and we have reported that ovariectomized rats fed normal chow show higher spine density in CA1 and prefrontal cortex pyramidal neurons and perform better on the object placement task than subjects fed a low phytoestrogen diet [20]. Because aged females have low circulating estradiol levels in general [33, 34, 41], dietary phytoestrogens may attenuate age-related declines in spatial memory and/or neural morphology. Young rats would not be expected to benefit from phytoestrogens because their estrogen levels are not low over the entire estrous cycle. Most aging studies utilize typical rat chow which contains high levels of phytoestrogens. Other factors which may have influenced outcomes in this study are enrichment, handling, stress, and the time that the animals were killed after behavior which may alter dendritic spine density. The current subjects received extensive handling and habituation to the spatial memory task in order to mitigate possible interfering effects of stress on memory and neural function [33]. In addition, the object placement task which was utilized here does not require either positive or negative reinforcements like the water maze and radial arm maze. These reinforced tasks can be stressful as evidenced by increased corticosterone levels [42]. Finally, the current subjects did not receive enriched housing or experience which can mitigate aging effects [12, 43, 44].

In conclusion, we found that a decrease in hippocampal CA1 dendritic spine density in aged female rats may contribute to poorer spatial memory as compared to young rats, but it is important to note that these results may only pertain to female subjects having had a specific set of life experiences. Specifically, these females did not have any reproductive experience, enriched housing or experiences, had a low phytoestrogen diet, and experienced minimal stress during the experiment. Since this age-related decrease in spine density is contrasted with results from other studies, it suggests that environmental variables may exert neuroplastic effects on aging; however, this hypothesis clearly requires further testing. Whether life experiences and diet may influence aging in humans is largely unknown but they could be important variables for investigation.

## Abbreviations

OVX: Ovariectomized
T1: Sample trial
T2: Recognition trial.
